# A cross-sectional study on tobacco use and dependence among women: Does menthol matter?

**DOI:** 10.1186/1617-9625-10-19

**Published:** 2012-11-27

**Authors:** Judith Rosenbloom, Vaughan W Rees, Kathleen Reid, Jeannie Wong, Taru Kinnunen

**Affiliations:** 1 Tobacco Dependence Treatment & Research, Department of Oral Health Policy and Epidemiology, Harvard School of Dental Medicine, Harvard Medical School, 188 Longwood Avenue, Boston, MA, 02115, USA; 2 Total Health Care, 1501 Division Street, Baltimore, MD, 21217, USA; 3 Center for Global Tobacco Control, Department of Society, Human Development and Health, Harvard School of Public Health, 677 Huntington Ave, Boston, MA, 02115, USA; 4 Department of Psychiatry, Boston Medical Center, 771 Albany Street, Dowling 7103, Boston, MA, 02118, USA; 5 Oliver Wyman, Health & Life Sciences, 1166 Avenue of the Americas, New York, NY, 10036, USA

**Keywords:** Cigarette smoking, Menthol cigarettes, Tobacco use, Tobacco dependence, Race/Ethnicity, Female Sex/Gender

## Abstract

**Background:**

The question of whether mentholation of cigarettes enhances tobacco dependence has generated conflicting findings. Potential mediating factors in a putative relationship between menthol use and tobacco dependence may include race and gender. While an association between menthol use and dependence is mixed, research on the role of race solely among women smokers is scarce. This study examined whether women menthol smokers have higher tobacco use and dependence than non-menthol smokers. Further, the study investigated differences between White and African American smokers.

**Methods:**

A cross-sectional study was conducted among 928 women seeking tobacco dependence treatment in Boston, Massachusetts. Measures obtained included preferred brand and menthol content, dependence markers (cigarettes per day (CPD); time to first cigarette in the morning; number of and longest previous quit attempts) and smoking history (age of initiation; years smoking; menthol or non-menthol cigarette preference). Analysis of variance (ANOVA) was used to detect interactions between menthol preference by race for continuous variables, and Pearson’s chi-squared test was used for analyses with dichotomous variables.

**Results:**

A greater proportion of menthol smokers smoked their first cigarette within five minutes of waking (p < 0.01) and were less likely to have a previous quit attempt longer than 90 days (p < 0.01). ANOVAs revealed no main effects for menthol preferences. However, African American smokers smoked fewer CPD (p<.001), started smoking later in life (p= .04), and had been smoking the same brand for longer (p= .04).

**Conclusions:**

Women menthol smokers showed signs of greater tobacco dependence than non-menthol smokers. African Americans smoked fewer CPD but nevertheless had evidence of greater dependence.

## Introduction

In the cigarette market in the United States, one quarter of cigarettes are labeled “menthol”. Over 70% of African American smokers use menthol cigarettes, compared with 30% of White smokers [1,2]. Further, menthol cigarettes are disproportionately preferred by adolescents, adult women, and those with low income [1-3].

Menthol as an additive to cigarettes has been a topic of scientific and public health attention for the past 20 years. As a result of the Master Settlement Agreement in 1998 between five major tobacco companies in the U.S. and Attorneys General in 46 states, tobacco industry internal documents regarding product design and development have become available to researchers and have shed light on the effect of cigarette additives. Menthol is hypothesized to be a factor that contributes to higher dependence and increased difficulty in quitting. This may lead to a higher lifetime exposure to tobacco and its by-products and a subsequent higher prevalence of tobacco-related morbidity and mortality. Review articles that have looked at the health effects comprehensively suggest that the relevant biological systems, namely the cardiovascular and respiratory systems, may be themselves affected by the menthol additive [4,5].

The significance of menthol as a cigarette additive was recognized in 2009 with *The Family Smoking Prevention and Tobacco Control Act,* giving the Food and Drug Administration (FDA) the authority to regulate tobacco products [6]. The Tobacco Products Scientific Advisory Committee for the FDA has stated that menthol is not simply a flavoring additive. Because of its ability to mask smoke harshness and irritation from nicotine, menthol has become a popular cigarette choice, especially among African Americans and youth. While menthol cigarettes are not widely used in Europe, the EU Scientific Committee on Emerging and Newly Identified Health Risks is also reviewing menthol as a harmful additive in tobacco products.

While there are no current regulations in place regarding menthol, research has continued to point to its contribution to tobacco use initiation, dependence and related morbidity. *Tobacco Induced Diseases* published a supplement in May 2011, consisting of seven papers reviewing the literature. It concluded that menthol is a biologically active compound which has primary actions, as well as secondary effects in conjunction with nicotine. However, much of the data on related topics were inconclusive [3,4,7-11].

Some studies have shown African Americans to be more nicotine-dependent than White smokers [12-15]. African Americans have lower cessation rates even after accounting for socioeconomic and demographic factors [16]. One study found that African Americans are more likely than Whites to report both strong motivation to quit and unsuccessful quit attempts in the past year [12], suggesting that they experience more difficulty quitting. This study also found that African Americans are more likely to smoke their first cigarette within ten minutes of waking up, indicating greater tobacco dependence. Finally, despite lighter smoking patterns, African Americans have higher blood serum levels of cotinine, a nicotine metabolite that serves as an indicator of nicotine exposure and dependence [13,17-19]. Research specifically on cigarette mentholation and tobacco dependence, independent of race, is limited and has generated mixed results. To better understand the relationship between tobacco use and dependence, other measures in addition to CPD, such as time to first cigarette, number and length of previous quit attempts, and salivary or blood biomarkers, need to be assessed [15,20].

When gender is considered as a factor, the data on menthol cigarettes, dependence, and morbidity becomes even less clear, as women smokers in the US are more likely than male smokers to choose menthol [2,3,21]. Further research examining the relationship between salivary cotinine levels and CPD among African Americans, Whites, men and women has shown differences between the races and genders. Kinnunen and colleagues found a positive correlation between salivary cotinine levels and CPD among men and women, both African American and White, but this relationship was restricted to non-menthol smokers. Among menthol smokers, there was only a significant positive correlation between salivary cotinine and CPD for White men. Thus, menthol may have a differential effect on smokers depending on their race and gender [15,20].

Research around tobacco use and dependence is important because tobacco-attributable illnesses account for approximately 440,000 deaths in the United States [22] and 5.4 million deaths worldwide each year [23]. Tobacco use is the leading preventable cause of morbidity and mortality. In the United States, African Americans bear a disproportionate burden of smoking-related illnesses. It has been speculated that cigarette mentholation may increase health risks by enhancing tobacco dependence in menthol smokers [15,24-26]. Even though African Americans smoke fewer cigarettes per day than Whites, they have higher rates of tobacco-related cancers, heart disease, and stroke [14,27,28] and may be more nicotine-dependent [12-15]. Alternatively, menthol may affect the metabolism of nicotine and other cigarette smoke constituents, thus altering smoking habits or the toxicity of cigarette compounds [29]. Although menthol itself may be broken down into harmful products during combustion [29-31], one review of the scientific literature concluded that cigarette mentholation confers only limited toxicological effects that do not increase smoking-related health risks [32]. A systematic review regarding lung cancer found that among African American male smokers, lung cancer could not be attributed to menthol cigarettes [5]. In contrast, another review suggested that cardiovascular health outcomes may be worse for those smoking menthol cigarettes [4]. More specific examination of menthol and its effects among women and their health issues is warranted.

The aim of this study was to determine whether women menthol smokers have higher tobacco dependence than women non-menthol smokers, while accounting for the influence of White and African American race.

## Methods

A cross-sectional study was conducted among women smokers who were interested in participating in a randomized controlled trial (RCT) involving exercise and nicotine replacement therapy (nicotine patch) in Boston, Massachusetts. Data were collected between January 2004 and December 2007. There were a total of 1439 callers of which 1412 provided some information about their smoking behavior. Eligibility for the RCT was assessed for 1287 participants who were recruited by newspaper, radio and subway advertisements in addition to postings on community bulletin boards [33]. The study was approved by the Harvard Medical School Office for Research Subject Protection (August 2003, Protocol number M10951-109). A verbal consent was obtained from each caller. Providing any or no answer was voluntary, and each participant was informed that the data collected could be used in a publication but only in an aggregate manner.

Prospective study participants who called an advertised toll-free number were read a description of the study and asked to complete the screening questionnaire to determine eligibility for the parent RCT. All callers, including those not interested or eligible for the parent RCT, were asked to complete the screening questionnaire. The telephone screen required participants to report various information about demographic variables, smoking and quitting history, current tobacco use, as well as medical and psychological health history**.** For the current analysis we included the participants who provided information regarding menthol smoking status, race, tobacco use and dependence measures (n=928).

Tobacco dependence was measured with six self-reported markers: number of cigarettes per day, age started smoking, length of time smoking, current brand, number of previous quit attempts, the time to first cigarette in the morning (TTF) (5 min or less; more than 5 min) [34,35], and the longest previous quit time (LQT) (90 days or less; more than 90 days) [36].

Separate analyses of variance (ANOVA), with independent variables menthol and race, were conducted with continuous variables age, CPD, age started smoking, years smoking current brand, and number of quit attempts. Pearson’s Chi squared test was used to analyze the categorical variables of dependence (% LQT > 90 days, % TTF < 5 min). Tukey’s HSD for unequal sample sizes was used for post hoc analysis.

## Results

A total of 198 African American and 730 White smokers provided data both for race and menthol status. Mean age for this sample was 40.9 (10.1) years and CPD was 18.1 (8.0) cigarettes. The results for age and six tobacco use measures by menthol status and race are provided in Table 1. For age, there was a significant (p=.04) interaction between *menthol preference* and *race* suggesting that the African American non-menthol smokers were relatively older 44.3 ± 10.7. There were no significant differences in the post hoc comparisons.

**Table 1:** Demographic, tobacco use and dependence measure by menthol and non-menthol smokers (M ± SD)

**Current age (y)**	**Menthol**	**Non-Menthol**	**p-value**
	**n = 335**	**n = 593**	
All (**n = 928**	41.1 ± 10.1	40.9 ± 10.1	.20^a^
White (**n = 730)**	41.6 ± 9.9	40.7 ± 10.1	.04^b^
AA (**n = 198**	40.5 ± 10.2	44.3 ± 10.7	
**Cigs Per day (CPD)**			
All	16.8 ± 7.4	18.8 ± 8.2	.72^a^
White	18.7 ± 7.4	19.1 ± 8.1	.40^b^
AA	14.9 ± 6.9	13.9 ± 7.9	
**Age started smoking (y)**			
All	16.7 ± 5.8	16.1 ± 4.1	.76^a^
White	16.5 ± 6.1	16.0 ± 4.1	.23^b^
AA	17.0 ± 5.4	17.7 ± 5.3	
**Years smoking current brand**			
All	14.5 ± 10.0	13.2 ± 10.1	.27^a^
White	13.4 ± 9.8	13.1 ± 10.2	.34^b^
AA	15.7 ± 10.0	12.9 ± 6.9	
**# Quit attempts**			
All	3.1 ± 3.2	3.3 ± 3.0	.81^a^
White	3.2 ± 3.3	3.3 ± 3.0	.98^b^
AA	3.0 ± 3.1	3.0 ± 3.9	
**Time To First Cig** (N = 928)	%	%	
All <5min	49.0 %	36.0 %	< .001^c^
n=928 >5min	51.0 %	69.0 %	
White <5min	39.5 %	35.7 %	.36^c^
n=730 >5min	60.5 %	64.3 %	
AA <5min	58.3 %	40.0 %	.06^c^
n=198 >5min	41.7 %	60.0 %	
**Longest Quit Time** (months; n=928)			
All <90 days	56.0 %	46.5 %	.01^c^
>90 days	44.0 %	68.3 %	
White <90 days	46.6 %	53.7 %	.11^c^
>90 days	53.4 %	46.3 %	
AA <90 days	58.2 %	42.8 %	.13^c^
>90 days	41.8 %	57.2 %	

^*a*^*Menthol preference* main effect; ^b^*Menthol preference* x *Race* interaction; ^c^ Pearson Chi-square.

The ANOVAs conducted showed no significant *menthol preference* main effects on cigarettes per day, age of smoking initiation, number of years smoking current brand, and number of quit attempts. However, significant *race* main effects were found. African American smokers smoked fewer CPD (p< .001), started smoking at an older age (p = .04) and had smoked the same brand for a greater length of time than White smokers.

Menthol smokers showed evidence of greater tobacco dependence than non-menthol smokers. We found that a larger percentage of the menthol smokers (49.0%) than the non-menthol smokers (36.0 %) reported smoking their first cigarette within 5 minutes of waking up in the morning (*p* <*.*01); and fewer of the menthol smokers (44.0%) than the non-menthol smokers (68.3%) reported a previous quit attempt of greater than 90 days (*p* <*.*01).

## Discussion

Among women smokers seeking tobacco dependence treatment, a significant difference according to menthol use status was observed on two measures of tobacco dependence: time to first cigarette in the morning and longest previous quit attempt. We found that more of the menthol smokers, as compared to the non-menthol smokers, reported smoking their first cigarette within 5 minutes of waking up in the morning. Additionally, fewer menthol smokers reported a previous quit attempt of greater than 90 days. The data suggest that, among women smokers, menthol cigarette use is associated with higher tobacco dependence. These findings compare closely with an earlier study of women smokers which showed that menthol smokers had a shorter time to first cigarette in the morning than non-menthol smokers [37]. Another study found that smokers of menthol cigarettes were less likely to make quit attempts and more likely to relapse as compared to non-menthol smokers [26]. When comparing race, an observational study found that, on average, African American smokers reported a shorter time to first cigarette in the morning [17]. Menthol was not evaluated separately in this analysis but was reported as a possible mitigating factor.

On the other hand, a prospective study of smoking cessation found no association between cigarette mentholation and various measures of nicotine dependence, including smoking cessation, number of cigarettes per day, and time to first cigarette [15]. Another study found that although African Americans are less likely to quit, smoking menthol cigarettes did not affect cessation rates in either race [25]. A recent review article concluded that menthol smokers are more nicotine dependent when using certain markers, such as time to first cigarette in the morning and night waking to smoke. However, when using other markers for dependence, such as cigarettes per day, this was not true [8]. These mixed results suggest that more definitive research is needed, preferably using biochemical verification in addition to self-reported measures.

Despite the observation of fewer cigarettes smoked per day, higher measures of dependence were observed among menthol smokers. Two potential mechanisms may be used to account for the higher tobacco dependence. Menthol may facilitate greater absorption of cigarette smoke toxins by allowing for deeper, prolonged inhalation and larger puffs [14,29,31], or by increasing the permeability of respiratory membranes [29,38]. Menthol imparts a soothing, “cooling” sensation by stimulating cold receptors in the respiratory tract [14,39]. As a cigarette additive, menthol may mask the irritation of cigarette smoke upon inhalation. Differences in tobacco dependence between menthol and non-menthol smokers should be investigated further to establish whether menthol encourages initiation of tobacco use, especially among youth, as well as further explore the relationship between menthol and tobacco dependence.

Several methodological limitations must be acknowledged. The sample was based upon women from an urban area of the northeastern region of the United States who were interested in quitting smoking, and therefore may not represent all smokers. The cross-sectional design also limits conclusions regarding the nature of the relationship between menthol preference and dependence. In addition, the data for tobacco dependence and morbidity were self-reported, and thus subject to recall or reporting biases. Future studies may use blood cotinine levels, a nicotine metabolite, to measure tobacco exposure. Socioeconomic status was not included as a factor in this analysis. Future research should include this element as lower socioeconomic status has been linked with increased markers of tobacco dependence [40]. Finally, regression analyses, which would simultaneously assess the influence of multiple independent variables on tobacco dependence outcomes, might offer a more definitive interpretation of the data. This approach was not used owing to small cell sizes for African American, non-menthol smokers.

## Conclusions

Women menthol smokers show increased evidence of tobacco dependence, including smoking their first cigarette within five minutes of waking and having a history of shorter previous quit attempts. This study confirmed previous research that African Americans, who are more likely than Whites to smoke menthol cigarettes, smoke fewer cigarettes per day. This suggests that menthol smokers may find it more difficult to quit smoking and specifically African Americans who predominantly smoke menthol cigarettes. Since there are relatively few previous studies looking at menthol, race, health and tobacco dependence disparities, this foundational data is an important basis for future research. However, more research is needed to identify the exact relationship between race and menthol cigarettes in order to create targeted successful cessation programs.

## Competing interests

The authors declare that they have no competing interests.

## Authors’ contributions

JR participated in the design of the study, the data collection and analysis, and the drafting of the manuscript. VR participated in the data analysis and the drafting of the manuscript. KR participated in the design of the study and the data collection. JW participated in the data collection and drafting of the manuscript. TK conceived of the study, acquired funding and participated in its design and coordination, data analysis and drafting of the manuscript. All authors read and approved the final manuscript.

## Authors’ information

The present work was conducted as a student at the Harvard School of Dental Medicine.
